# Notes and News

**Published:** 1897-02-13

**Authors:** 


### Notes and News.

(Collected and Communicated.)

Mr. C. J. Thomas has been appointed chairman of the Metropolitan Hospital in place of the late Mr. Joseph Fry.

No less than seven unregistered dentists have recently appeared in the Brighton police courts, accused of having practised under false pretences. All were convicted, and fines varying from £2 to £10, with costs, were imposed.

No better news comes from Bombay respecting the plague. Some of the worst infected houses have been burnt, but the complications which attend the disease, such as pneumonia, cause many cases to pass unrecorded under their true heading, and increase the difficulties of the situation. The infected districts are gradually increasing; they now include Poona, Karachee, and Kandahar; whilst the Portuguese district south of Bombay is threatened. The authorities have adopted stringent police regulations to prevent its introduction into new districts, but very inefficient sanitary precautions have been taken.

The Director-General of the Army Medical Department, when addressing the students at Netley at the close of the recent session, alluded to disabilities under which the army medical service laboured, but remarked that army surgeons had from time to time given rise to irritation by a habit of assuming their military rank apart from its medical connection. They were also accused of adopting the practice of writing anonymously to the Press, a course not redounding to the credit of the service.

Two new wings have just been opened at the Gorleston Cottage Hospital, Great Yarmouth, by Colonel H. Buxton, the Mayor. The hospital was commenced with two beds in a hired house. Later a site was given by Mr. Hervey-George, and recently the munificent sum of £1,000, bequeathed by Miss Harriet Miller, enabled an extension to be made. The two wings contain male and female wards, each with five beds, the necessary offices, and an operating-room. A covered way has been erected to the out-patients department, and many other additions and improvements have been made.

The Indian Famine Fund at the Mansion House equals upwards of £250,000.

The Lord Mayor will preside at a meeting on behalf of the Charing Cross Hospital to be held at the Mansion House on Friday, March 5th, at three p.m.

Dr. G. H. Bristow has been elected Senior House Surgeon at the Johannesburg Hospital. Dr. Bristow was formerly House Physician at the Brompton Consumption Hospital.

The use of collecting boxes in connection with the Royal Hospital at Richmond is very extensive. The boxes number about three hundred, and during the past year £658 were added to the funds of the hospital by this means.

By command of H.R.H. the Princess of Wales, the sum of £3 7s. 1d. has been forwarded to the British Home for Incurables, Crown Lane, Streatham, being a further portion of part proceeds of the sale of Canon Fleming's sermon, "Recognition in Eternity."

The Bishop of Chichester will open the Rustington Convalescent Home at Littlehampton on March 20th. The institution has been erected and endowed by Mr. Henry Harben, J.P., for the working-classes of North-West London. The munificent donation represents £70,000.

A grand festival banquet in aid of the funds of the Great Northern Central Hospital will be held at the Hotel Cecil on May 11th, for the purpose of celebrating the sixtieth year of Her Majesty's reign by raising the sum of £20,000 to enable the two remaining unused wards to be opened. Mr. B. L. Cohen, M.P. for East Islington, has consented to take the chair.

Rather a novel departure has been taken at the Charité Hospital, Berlin. This is the establishment of a department for accidents only, where the patients will be retained until their complete restoration to health. Medical baths and apparatus will be provided to further recovery. The patients will be expected to pay three shillings per diem. Members of trade unions will be allowed to receive the visit of the doctor of their association in conjunction with the visiting staff, who will thus be able to watch the treatment and progress of cases sent in by them.

The inquiry into the result of the present muzzling regulations for dogs is quite startling in its conclusions. As the Board of Agriculture reports, the orders as enforced since 1892 had caused the maximum of irritation and the minimum of efficiency. From 1889 to 1892 the regulations of the Board of Agriculture were enforced with the result that in 1889 there were 312 cases of rabies, and in 1892 the number was reduced to 38. After that date the matter fell into the hands of the local authorities, with the result that, in 1895, no less than 672 cases were reported, notwithstanding the fact that thousands of stray dogs were put down each year, and that thus the principal source of danger was removed. It is disheartening for the owners of dogs, who have been patiently hoping for the emancipation of their favourites, and the ultimate freedom from danger both for the animals and the public, to learn that, from faulty methods of application, all has been worse than fruitless, and that we are worse off than in 1892.

